# Utilizing bifurcated allogeneic vein grafts: a novel approach for preventing sinistral portal hypertension following pancreaticoduodenectomy. A 10-year before and after study

**DOI:** 10.1097/JS9.0000000000001944

**Published:** 2024-07-12

**Authors:** Jing Wang, Shao-cheng Lyu, Song-ping Cui, Jin-can Huang, Han-xuan Wang, Bin Hu, Qiang He, Ren Lang

**Affiliations:** a Department of Thoracic Surgery, Beijing Institute of Respiratory Medicine and Beijing Chao-Yang Hospital, Capital Medical University, Beijing, China; b Mass General Cancer Cennter, Mass General Brigham, Harvard Medical School; c Department of Hepatobiliary and Pancreaticosplenic Surgery, Beijing Chaoyang Hospital, Capital Medical University, Beijing, China

**Keywords:** bifurcated allogeneic vein, pancreatic carcinoma, prognosis, sinistral portal hypertension, vascular reconstruction

## Abstract

**Background:**

Sinistral portal hypertension (SPH) may occur in patients with pancreatic carcinoma after pancreaticoduodenectomy (PD) with spleno-mesenterico-portal (S-M-P) confluence resection. This study aimed to evaluate outcomes with bifurcated allogeneic vein replacement in the prevention of SPH in pancreatic carcinoma patients.

**Materials and methods:**

A total of 81 patients were included. The authors retrospectively collected clinicopathological data from 66 patients underwent PD with S-M-P confluence resection in our hospital from January 2011 to December 2021, compared the correlation between different venous reconstruction methods using log-rank tests and clinical outcomes through univariate and multivariate analyses. Secondly, the authors prospectively collected clinical data and outcomes of 15 patients who underwent splenic vein reconstruction from January 2021 to January 2023.

**Results:**

In the retrospective study, 43 cases received reconstruction by bifurcated allogeneic vein (Reconstruction group) and 23 cases received simply SV ligation (Ligation group). The preoperative platelet counts and spleen volume were similar between two groups (*P*>0.05). Nevertheless, at 1 month, 3 months and 6 months after operation, the related indexes of SPH such as platelet count, spleen volume, spleen volume ratio and esophagogastric varices (EGV) grade in Reconstruction group were better than those in Ligation group (*P*<0.05). 6 months after surgery, the incidence of SPH in Ligation group was significantly higher than in Reconstruction group (36.4% vs. 8.1%, respectively). In the prospective study, the incidence of SPH in patients undergoing SV reconstruction was 6.7% (1/15).

**Conclusions:**

Without compromising surgical outcomes, reconstruction of the S-M-P confluence by bifurcated allogeneic vein is a better method to avoid SPH in patients with advanced pancreatic carcinoma.

## Introduction

Highlights

- Introduces a novel surgical method utilizing bifurcated allogeneic vein grafts to prevent sinistral portal hypertension (SPH) following pancreaticoduodenectomy with combined portal vein resection.
- Robust evidence from a decade-long study, including both retrospective and prospective data, supports the effectiveness of this technique.
- Demonstrates improved patient outcomes, including lower incidence of SPH, better platelet counts, reduced spleen volume, and improved varices grading.
- Enhances the safety of pancreaticoduodenectomy and potentially improves the quality of life for patients with advanced pancreatic cancer.

Early invasion of the portal system represents one of the most common characteristics of pancreatic head/uncinate process carcinoma^1,2^. Extensive pancreaticoduodenectomy (PD) with portal vein or superior mesenteric vein (PV/SMV) resection has been adopted by most surgeons for the treatment of pancreatic head/uncinate process carcinoma^3,4^. Extensive surgical operation offers the only potential cure for pancreatic cancer with PV/SMV invasion. A large proportion of patients can benefit from extensive resection on account of improvement of margin-negative rate, thus getting relatively longer postoperative survival^5^. Invasion of spleno-mesenterico-portal (S-M-P) confluence is deemed as a tricky problem for surgeons^6^. Improper treatment methods during operative procedure will be related to several complications after the operation. PV/SMV reconstruction with simply ligation of splenic vein (SV) is usually performed after resection of S-M-P confluence. However, sinistral portal hypertension (SPH), as one of the major complications, is frequently found in some patients for whom this method is applied.

SPH, which is also referred to as left-sided, segmental, regional, localized, compartmental, lineal, or splenoportal hypertension, is a localized form of portal hypertension frequently resulted from isolated obstruction of SV^7^. Patients’ health and quality of life is threatened by serious harms including splenomegaly, hypersplenism, varices and gastrointestinal bleeding, which result from SPH^8,9^. However, some surgeons assert that SV reconstruction represents unnecessary due to the left gastric vein or other collaterals are able to work as the venous outflow from the spleen, and SPH rarely causes clinically relevant consequences^10–13^. Nevertheless, these collateral veins do not have to be retained because they are close to the tumor, and recent researches claimed that patients will develop SPH within 6 months after surgery if none of the potential collateral veins is preserved^14^.

With the aim of avoiding SPH, various strategies have been carried out, for instance, simultaneous ligation of splenic artery, SV-SMV anastomosis, SV-inferior mesenteric vein (IMV) anastomosis, temporary mesocaval shunt with distal splenorenal shunt, and so forth^15–18^.

Here, we present our novel method for dealing with pancreatic head/uncinate process cancer with infiltration of S-M-P confluence. After completion of PD with an “en-bloc” resection of the S-M-P confluence, the confluence is reconstructed by using bifurcated allogeneic vein, which is from donation after cardiac death. Because the confluence is rebuilt during the operative procedure, SPH is avoided perfectly. With advancements in surgical techniques, our center initiated pancreatic cancer surgeries involving en-bloc resection and reconstruction of the portal system starting in 2011. As our surgical procedures progressed and techniques improved, we increasingly resected longer sections of invaded vessels, eventually transitioning to the use of branchless allograft veins for replacement. This technique has broadened the surgical indications at our center, allowing for the resection of tumors with an invasion length exceeding 5 cm. However, we observed that the splenic vein could not be preserved in patients with portal vein invasion and had to be severed. Initially, we simply disconnected the splenic vein without reconstruction, but follow-up revealed that some patients developed regional portal hypertension and even presented with gastrointestinal bleeding. Given the varied shapes and sizes of the donated allograft vessels we obtained, we considered using bifurcated allogeneic vein grafts for simultaneous reconstruction of the portal vein, superior mesenteric vein, and splenic vein, which has evolved into the current portal system reconstruction technique. Few reports have been touched upon this method, and the correlation between the treatment method and SPH have not yet been reported. The aim of the study is to introduce this novel method and to analyze the correlation between the method and SPH.

## Materials and methods

The work has been reported in line with the STROCSS criteria^19^, Supplemental Digital Content 1, http://links.lww.com/JS9/D98.

### Patient collection

#### Retrospective study

We retrospectively collected data from patients who underwent PD combined with portal venous confluence resection and reconstruction in our hospital from January 2011 to December 2021.

Inclusion criteria: (1) underwent PD and was judged that the S-M-P confluence invaded or could not be separated; (2) no invasion of the main abdominal arteries (abdominal trunk, common hepatic artery, abdominal aorta) or distant metastasis; (3) complete surgical resection of the tumor and the confluence of the portal vein system, and the method of vascular remodeling is not limited; (4) the spleen was not jointly removed during the operation; (5) perioperative survival; (6) clinical and follow-up data were complete.

Allogeneic blood vessels were collected by organ procurement organizations (OPO) in our hospital when obtaining donor organs. This study has been approved by the ethics committee and the clinical application management committee of medical technology of our hospital for clinical application.

#### Prospective study

“A prospective study” was approved and implemented by the ethics committee of our hospital in January 2020, and registered through the website of the China Clinical Trials Registry.

Inclusion criteria: (1) age 18–80 years with no gender restrictions; (2) preoperative imaging showed that the S-M-P confluence was invaded, and the SV needed to be reconstructed; (3) preoperative evaluation without distant metastasis or invasion of important abdominal arteries (abdominal trunk, common hepatic artery, abdominal aorta); (4) preoperative evaluation was unremarkable to surgery such as cardiopulmonary dysfunction; (5) expected survival was more than 6 months; (6) patients and their families agreed to the study protocol and signed the informed consent form.

Exclusion criteria: (1) preoperative presence of portal hypertension-related disease; (2) joint resection of the spleen during surgery; (3) SV reconstruction was not used by bifurcated allogeneic vessels; (4) The patient died within 6 months after surgery.

A total of 15 patients were planned to be enrolled in this study, which was completed in January 2023, and all patients were followed for at least 6 months.

### Portal vein system confluence reconstruction method

**Figure 1:**
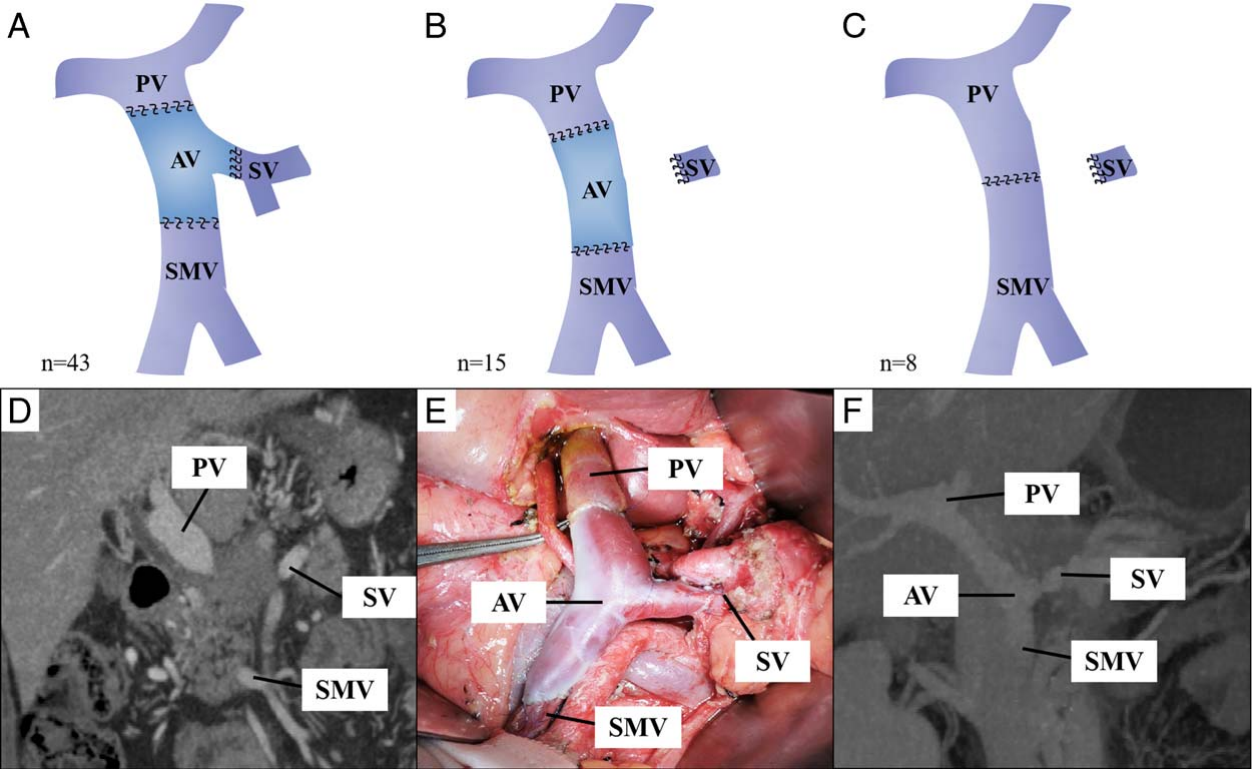
Reconstruction after resection of the portal vein system (*n*=66). (A) Pattern diagram of portal vein system reconstruction using bifurcated allogeneic vein. (B) Pattern diagram of portal vein-superior mesenteric vein reconstruction using tubular allogeneic vein and splenic vein ligation. (C) Pattern diagram of portal vein-superior mesenteric vein end-to-end anastomosis with ligation of splenic vein. (D) Preoperative computed tomography (CT) showed that pancreatic cancer invaded the confluence of the portal vein system; (E) Intraoperative photograph of bifurcated allogeneic vein reconstruction of portal vein-superior mesenteric vein-splenic vein. (F) CT at 3 months postoperative showed reconstructive vascular patency. AV: allogeneic vein; PV, portal vein; SMV, superior mesenteric vein; SV, splenic vein.

Patients were divided into Reconstruction group (*n*=43) and Ligation group (*n*=23) based on whether or not the SV was reconstructed. Reconstruction group: at least 1 cm from the edge of the tumor, the PV, the SV and the SMV were blocked, and the invaded confluence of portal vein system and the tumor were removed. Bifurcated allogeneic veins (Fig. 1A, D-F) were used to anastomosis reconstruction with PV, SV and SMV, respectively.

Ligation group: at least 1 cm from the edge of the tumor, block the PV, SV and SMV, and removed the invaded confluence of portal vein system and the tumor. The SV was directly ligated and the PV-SMV was reconstructed by tubular allogeneic vein (Fig. 1B) or direct end-end anastomosis (Fig. 1C) depending on the length of the PV.

Acquisition and use of allogeneic blood vessels: After allogeneic vascular acquisition, it was soaked in antibiotics (penicillin + streptomycin) for 24 h, and then stored at low temperature, and the storage period was 2 weeks^20^. Before using venous vessels, they need to be rewarmed at room temperature for 30 min^21^.

For patients undergoing PD at our center, It is routine not to preserve the pylorus of the stomach, leading to the direct division of the left gastric vein. In cases where the tumor has invaded the portal system, we also routinely divide the right gastroepiploic vein during the management of the gastrocolic trunk. Regarding the IMV, for preoperative patients considered to have vascular invasion, we commonly adopt an approach via the IMA to prioritize exploring arterial involvement, which involves the division of the IMV during this anatomical process. However, for other causes of PD, we preserve the IMV.

As for intraoperative vascular replacement procedure, Firstly, we use vascular clamps to respectively block the PV, SMV and SV. Then, sever the vessels more than 5mm from the tumor invasion margin, and excise the tumor along with the invaded vessels en bloc. Send the intraoperative frozen sections of the three severed vessels for pathological margin assessment. Meanwhile, take the cryopreserved allogeneic vessels and tailor them according to the vascular defect ex vivo (S-Fig. 1A-C, Supplemental Digital Content 2, http://links.lww.com/JS9/D99). After confirming negative margins from the intraoperative pathology analysis, proceed with the reconstruction using the allogeneic vessels.

To alleviate intestinal congestion and quickly restore intestinal blood flow, we prioritize the reconstruction of the SMV. Using 6-0 prolene sutures, perform continuous everting anastomosis of the anterior and posterior walls between the allogeneic vessel and the SMV. Then, perform a similar anastomosis between the allogeneic vessel and the PV. Release the vascular clamps on the PV and SMV to restore blood flow between them. Subsequently, perform continuous everting anastomosis of the anterior and posterior walls between the allogeneic vessel and the SV using 6-0 prolene sutures. Release the clamp on the SV to restore blood flow between the SV and the PV, thus completing the allogeneic vessel replacement (S-Fig. 1D, Supplemental Digital Content 2, http://links.lww.com/JS9/D99).

### Strategies for perioperative treatment and postoperative follow-up

All patients in this study required postoperative pharmacological anticoagulation to prevent thrombosis in the reconstructed veins. Depending on the recovery of postoperative coagulation indicators and the characteristics of the drainage fluid, anticoagulation should be started within 24 h after surgery if the patient is not at significant risk of bleeding. All patients had an abdominal vascular ultrasound within 48 hours of surgery to determine whether thrombosis was present at the site of the reconstructed vessel.

Postoperative follow-up strategy: once in the 1st and 3rd months after surgery; 1 time every 3 months for 2 years after surgery; and more than 2 years once every six months. The items of postoperative follow-up examination mainly included laboratory tests, abdominal contrast-enhanced computed tomography (CT) examination, and further gastroscopy if the patient’s CT showed esophagogastric varices (EGV).

### Collection of clinical information and outcomes

Patient’s general information, surgical information (revascularization method, portal system reconstruction time, portal system resection length, etc.) and perioperative information (postoperative pathology, complications, etc.) were collected. Portal system reconstruction time was defined as the time between PV blood flow being blocked and reopened. The diagnosis of postoperative pathology was interpreted by two experienced pathologists, and if there was any objection, a third pathologist would be asked to interpret it.

Postoperative complications were graded according to the Clavien–Dindo classification^22^. Drain amylase of more than three times serum amylase after the third postoperative day, as defined by International Study Group of Pancreatic Surgery (ISGPS), was defined as pancreatic fistula^23^. Platelet ratio defined as: repeat platelet count/preoperative platelet count × 100%. Spleen volume ratio defined as: re-examination of spleen volume/preoperative spleen volume × 100%.

Varicose veins caused by SPH were divided into esophagogastric, perisplenic, and pericolonic. The degree of EGV refers to the criteria for endoscopic diagnosis by Chinese Society of Digestive Endoscopy (CSDE) and British Society of Gastroenterology (BSG)^24^.

The image processing system Advantage Workstation 46 (AW46) was used to measure the spleen volume. In addition to preoperative measurement, spleen volume was also measured 1 month, 3 months and 6 months after surgery. Spleen volume ratio was calculated as post-operation volume/pre-operation volume.

### Statistical analysis

Measurement data fitting normal distribution were expressed as mean±standard deviation while data fitting non-normal distribution were expressed as median (interquartile range). *t*-test was adopted for normal distribution and rank sum test was used for non-normal distribution when comparing measurement data between the two groups. χ^2^ test was used to compare the counting data between two groups and Fisher’s exact probability method was used when the theoretical frequency was less than 1. The incidence of SPH was compared using the Kaplan–Meier curves and log-rank tests. Statistical significance was defined as *P* less than 0.05, and all data were analyzed by SPSS 24.0 software.

## Results

### Retrospective study

#### Basic perioperative information

The retrospective study included a total of 66 patients, of whom 35 were female and 31 were male. The male-to-female ratio was 1:1.1 and the age was 58.4±11.7 years (range 29–79 years). All patients successfully underwent the resection and reconstruction of the portal vein confluence, with an average intraoperative bleeding volume of 600 (400–100) ml, blood transfusion in 44 cases (66.7%), operation time of 11.0±2.6 h, portal vein system resection length of 4.7±1.1 cm, portal vein system reconstruction time of 37.5±11.1 min.

Postoperative pathology: 49 cases of pancreatic cancer, 5 cases of distal cholangiocarcinoma, 4 cases of pancreatic neuroendocrine tumor, 4 cases of pancreatic solid pseudopapilloma, 2 cases of pancreatic serous cystadenoma, and 2 cases of mass pancreatitis. There were 23 cases of postoperative complications in all patients, and the complication rate was 34.8%. The main complications included: 7 cases of abdominal infection (10.6%), 5 cases of biochemical fistula (7.6%), 3 cases of grade B pancreatic fistula (4.5%), 2 cases of grade C pancreatic fistula (3.0%), 5 cases of gastric emptying disorder (7.6%), 5 cases of diarrhea (7.6%), 3 cases of abdominal bleeding (4.5%), and 2 cases of portal vein thrombosis (3.0%).

According to whether or not to reconstruct the SV, it was divided into Reconstruction group (n=43) and Ligation group (*n*=23). A comparison of perioperative data between the two groups was shown in Table 1. Compared with the Ligation group, the Reconstruction group only had a longer reconstruction time for the portal venous system than the control group (*P*<0.001), and there were no significant differences in other aspects.

**Table 1:** Comparison of perioperative data between the two groups (*n*=66).

Item	Reconstruction group (*n*=43)	Ligation group (*n*=23)	*P*
Sex (m/f)	21/22	10/13	0.678
Age (year)	57.3±12.1	60.4±11.1	0.298
Diabetes (Y/N)	14/29	8/15	0.855
Smoking history (Y/N)	10/33	6/17	0.798
White blood cells (×10^9^/l)	6.0±1.7	6.0±1.7	0.972
Hemoglobin (g/l)	115.3±19.6	113.7±22.3	0.767
Platelets (×10^9^/l)	228.9±97.6	242.8±85.2	0.566
Albumin (g/l)	37.1±6.6	36.7±5.7	0.807
Total bilirubin (μmol/l)	60.1 (10.5, 145.3)	72.8 (14.4, 237.1)	0.267
CEA (ng/ml)	2.5 (1.9, 4.7)	2.2 (1.8, 3.4)	0.531
CA19-9 (U/ml)	495.1 (44.3, 1161.9)	202.6 (44.4, 433.4)	0.297
Spleen volume (ml)	176.1±36.7	177.7±20.9	0.822
Neoadjuvant chemotherapy (Y/N)	5/38	2/21	0.959
Intraoperative blood loss (ml)	700 (500, 1000)	600 (400, 1000)	0.261
Intraoperative blood transfusion (Y/N)	29/14	15/8	0.855
Duration of surgery (h)	11.3±2.7	10.5±2.4	0.262
Portal vein system resection length (cm)	4.7±1.1	4.7±1.3	0.789
Portal venous system reconstruction time (min)	44.4±5.8	24.7±5.8	<0.001
Pathology (pancreatic cancer/other)	33/10	16/7	0.525
Tumor diameter (cm)	4.0±1.6	3.5±1.1	0.196
Lymph node metastases (Y/N)	28/15	12/11	0.305
Length of hospital stay after surgery (day)	20 (15, 25)	21 (16, 28)	0.762
Postoperative chemotherapy (Y/N)	13/30	3/20	0.121
Perioperative complications	14	9	0.593
Clavien–Dindo			0.915
I	6	4	
II	5	3	
III	2	2	
Biochemical fistula	2	3	0.460
Pancreatic fistula			
Grade B	2	1	0.573
Grade C	1	1	1.000
Gastric emptying disorders	3	2	0.813
Diarrhea	4	1	0.813
Abdominal infection	5	2	0.959
Abdominal bleeding	1	2	0.573
Portal vein thrombosis	1	1	1.000

CA19-9, carbohydrate antigen 19-9; CEA, carcinoembryonic antigen; f, female; m, male.

#### Long-term follow-up outcomes

**Figure 2:**
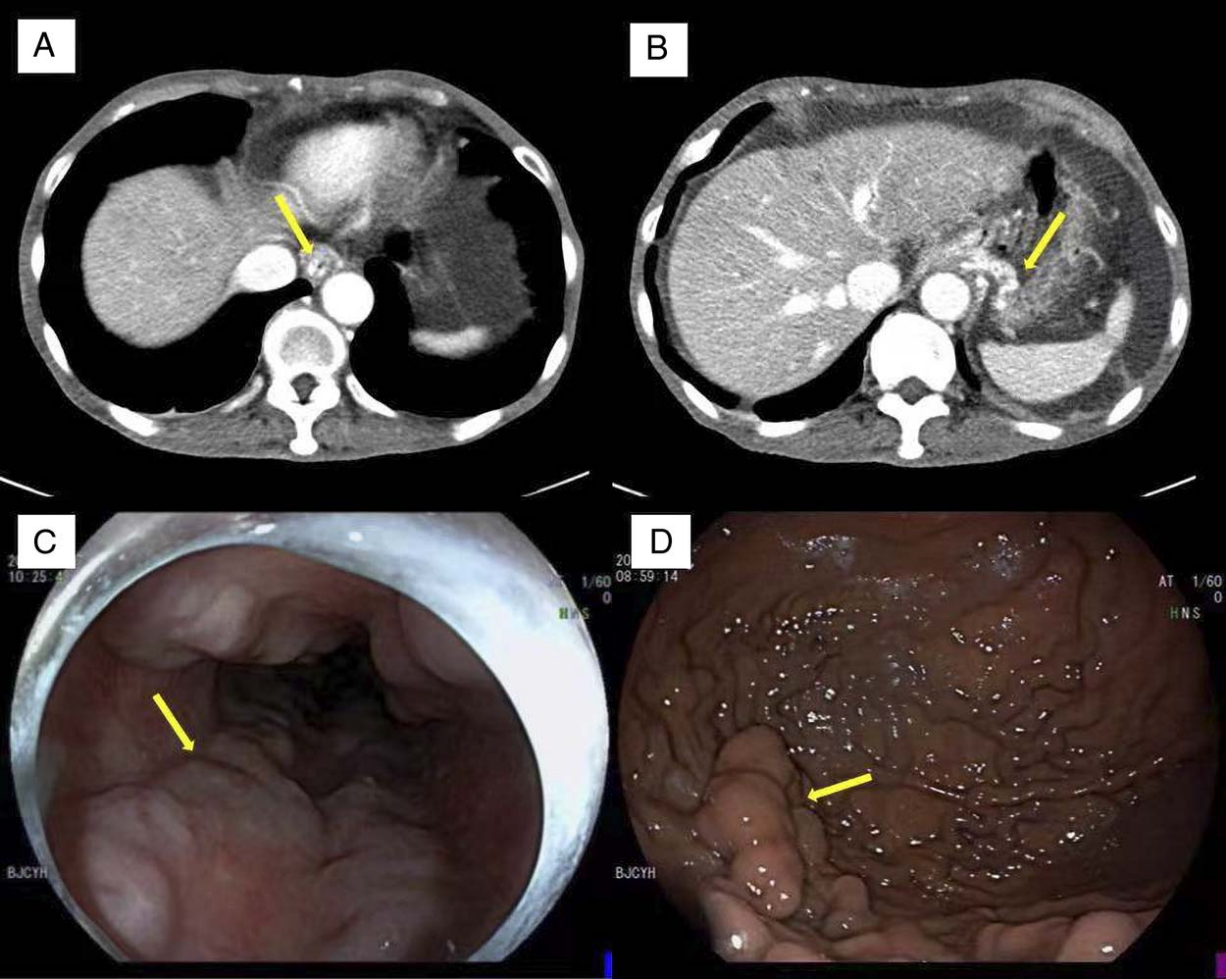
Varicose veins in patients at long-term follow-up. (A) Abdominal computed tomography (CT) showed a marked increase in the diameter of the lower esophageal vein. (B) Abdominal CT showed that the fundal veins were excessively tortuous and markedly thickened. (C) Gastroscopy showed four blue dilated blood vessels on the inner wall of the esophagus, and cherry red changes were visible on the surface of the vessels. (D) Gastroscopy shows nodular, beaded varicose veins in the stomach wall.

**Figure 3:**
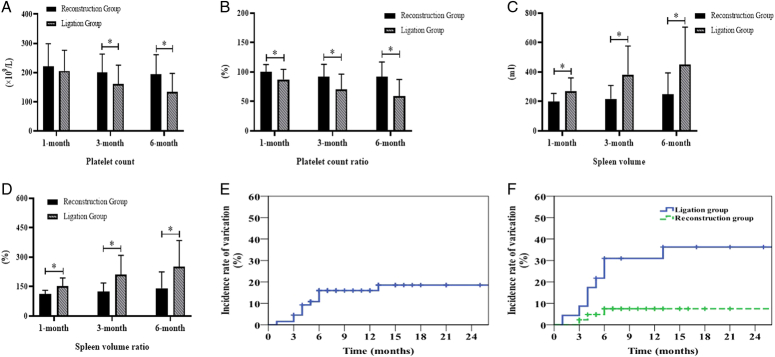
Long-term follow-up of patients in both groups (Reconstruction group=43, Ligation group=23). (A) Comparison of platelet counts in the two groups. (B) Comparison of platelet ratios between the two groups. (C) Comparison of spleen volume in the two groups. (D) Comparison of the proportion of spleen volume in the two groups. (E) Curve of varicose vein occurrence in all patients. (F) Curves of varicose vein occurrence in both groups of patients.

The last follow-up was December 2023, with a median follow-up of 18 months. The primary endpoint was varicose veins or death. During the follow-up period, a total of 3 patients in the Reconstruction group developed varicose veins, and no patients had variceal bleeding. A total of 8 patients in the Ligation group developed varicose veins and 2 patients developed variceal bleeding. The platelets, platelet ratio, spleen volume, spleen volume ratio and SPH in the Reconstruction group and Ligation group were detailed in Table 2 and Figure 2. Compared with the Reconstruction group, the platelet count and proportion of platelet count in the Ligation group was lower at all postoperative time points (*P*<0.05), while the spleen volume and proportion of spleen volume was significantly higher (*P*<0.05). At the same time, the incidence of SPH in the SV Ligation group at 6 months after surgery was higher than in the Reconstruction group (*P*<0.05) (Fig. 3). The incidence of SPH in this group was 1.5%, 4.5% and 16.7% at 1 month, 3 months and 6 months after surgery. The incidence of SPH in the Reconstruction group and Ligation group was 0%, 2.3%, 7.0% and 4.3%, 8.7%, 34.8% at 1 month, 3 months and 6 months after surgery, respectively.

**Table 2:** Comparison of postoperative follow-up data between the two groups (*n*=66).

	1 month		3 months		6 months	
Item	Reconstruction group (*n*=43)	Ligation group (*n*=23)	Reconstruction group (*n*=43)	Ligation group (*n*=23)	Reconstruction group (*n*=43)	Ligation group (*n*=23)
Platelets (×10^9^/l)	222.4±76.6	205.9±70.5	200.2±63.2	161.0±64.6^a^	195.1±66.3	133.7±63.4^a^
Platelet ratio (%)	99.9±12.7	87.3±17.1^a^	92.0±21.1	70.1±26.1^a^	92.2±24.8	58.6±28.7^a^
Spleen volume (ml)	176 (168, 209)	255 (207, 309)^a^	186 (168, 225)	336 (241, 458)^a^	188 (168, 255)	346 (266, 663)^a^
Spleen volume ratio (%)	106.7 (102.1, 111.5)	143.3 (113.1, 178.4)^a^	107.3 (102.1, 120.6)	172.3 (136.5, 274.4)^a^	111.9 (102.9, 130.3)	182.9 (150.7, 378.0)^a^
SPH	0	1	1	2	3	8^a^
Esophagogastric	0	0	1	1	2	5
Perisplenic	0	1	0	1	0	1
Pericolonic	0	0	0	0	1	2
EGV	0	0	1	2	2	5
Grade I	0	0	0	1	1	1
Grade II	0	0	1	0	1	2
Grade III	0	0	0	1	0	2
Esophageal variceal bleeding	0	0	0	1	0	2

The cases represent cumulative incidences.

EGV, esophagogastric varices; SPH, sinistral portal hypertension.

^a^ Indicates *P*<0.05 compared to Reconstruction group.

#### Risk factor analysis of SPH

According to whether SPH occurred after surgery, patients were divided into SPH group (*n*=11) and control group (*n*=55), and the risk factors for SPH were analyzed by logistic regression, as shown in Tables 3 and 4. The results showed that intraoperative non-reconstruction of the SV [odds ratio (OR)=19.050, 95% CI: 1.124–322.846] was the only independent risk factor for postoperative SPH.

**Table 3:** Univariate analysis for evaluating risk factors of SPH (*n*=66).

	Univariate analysis		
Item	SPH group (*n*=11)	Control group (*n*=55)	*P*
Sex (m/f)	6/5	25/30	0.581
Age (year)	58.4±7.7	58.4±12.4	0.999
Diabetes (Y/N)	3/8	19/36	0.907
Smoking history (Y/N)	5/6	11/44	0.158
White blood cells (×10^9^/l)	6.4±1.5	5.9±1.7	0.329
Hemoglobin (g/l)	124.9±24.0	112.7±19.2	0.070
Platelets (×10^9^/l)	229.4±96.4	234.6±93.2	0.866
Albumin (g/l)	38.0±6.6	36.7±6.3	0.557
Total bilirubin (μmol/l)	30.3 (13.8, 197.8)	65.2 (12.6, 149.8)	0.891
CEA (ng/ml)	2.4 (1.8, 4.0)	2.4 (1.9, 4.3)	0.816
CA19-9 (U/ml)	286.4 (105.0, 528.8)	243.0 (43.5, 1059.5)	0.938
Spleen volume (ml)	179.5±27.0	176.1±33.0	0.748
Neoadjuvant chemotherapy (Y/N)	1/10	6/49	0.721
Intraoperative blood loss (ml)	600 (450, 900)	600 (400, 1000)	0.612
Intraoperative blood transfusion (Y/N)	7/4	37/18	0.907
Duration of surgery (h)	10.5±2.3	11.1±2.7	0.447
Portal vein system resection length (cm)	4.9±1.0	4.7±1.2	0.433
Portal venous system reconstruction time (min)	31.3±13.2	38.8±10.3	0.039
Splenic vein reconstruction (Y/N)	3/8	40/15	0.011
Left gastric vein resection (Y/N)	10/1	54/1	0.308
Right gastroepiploic vein resection (Y/N)	10/1	51/3	0.533
Inferior mesentric vein resection (Y/N)	6/5	34/21	0.910
Pathology (pancreatic cancer/other)	10/1	39/16	0.314
Tumor diameter (cm)	3.3±1.0	3.9±1.5	0.189
Lymph node metastases (Y/N)	7/4	33/22	0.910
Postoperative chemotherapy (Y/N)	2/9	14/41	0.898
Perioperative complications	3/8	20/35	0.817

CA19-9, carbohydrate antigen 19-9; CEA, carcinoembryonic antigen; SPH, sinistral portal hypertension; f, female; m, male.

**Table 4:** Multivariate analysis for evaluating risk factors of SPH (*n*=66).

	Multivariate analysis	
Item	OR (95% CI)	*P*
Smoking history (Y/N)	0.295 (0.054–1.619)	0.160
Hemoglobin (g/l)	1.031 (0.988–1.075)	0.165
Portal venous system reconstruction time (min)	1.079 (0.935–1.244)	0.298
Splenic vein reconstruction (Y/N)	36.045 (1.194–1088.450)	0.039
Tumor diameter (cm)	0.646 (0.299–1.394)	0.266

OR, odds ratio; SPH, sinistral portal hypertension.

### Prospective study

#### Basic perioperative information

The prospective study included a total of 15 patients, including 9 males and 6 females, aged 63.6±9.8 years (range 48–78 years) (Table 5). All patients were successfully reconstructed at the confluence of the portal venous system by bifurcated allogeneic vessels, with average intraoperative bleeding of 800 (550–800) ml, blood transfusion in 7 cases (46.7%), operation time of 11.2±4.3 h, resection length of the portal venous system of 4.8±0.9 cm, and reconstruction time of portal venous system of 36.0±4.7 min.

**Table 5:** Baseline data and follow-up of patients (*n*=15).

Patient number	Sex	Age (year)	Pathology	Duration of follow-up	Survival situation	Varicosity	Site of varicose veins
No.1	Male	59	Pancreatic cancer	12	Alive	No	NA
No.2	Female	68	Pancreatic cancer	13	Dead	No	NA
No.3	Male	77	Cholangiocarcinoma	18	Alive	No	NA
No.4	Male	59	Pancreatic cancer	16	Dead	No	NA
No.5	Female	78	Pancreatic neuroendocrine tumors	9	Alive	No	NA
No.6	Male	48	Pancreatic cancer	9	Alive	Yes	Esophageal fundus
No.7	Male	77	Pancreatic cancer	10	Alive	No	NA
No.8	Female	68	Pancreatic cancer	8	Dead	No	NA
No.9	Female	63	Pancreatic cancer	7	Alive	No	NA
No.10	Male	56	Pancreatic cancer	7	Alive	No	NA
No.11	Female	52	Pancreatic cancer	6	Alive	No	NA
No.12	Male	65	Pancreatic solid pseudopapilloma	6	Alive	No	NA
No.13	Male	69	Pancreatic cancer	13	Dead	No	NA
No.14	Female	66	Pancreatic cancer	17	Alive	No	NA
No.15	Male	49	Pancreatic cancer	17	Alive	No	NA

NA, not applicable.

Postoperative pathology was as follows: 12 cases of pancreatic cancer, 1 case of distal cholangiocarcinoma, 1 case of pancreatic neuroendocrine tumor, and 1 case of solid pseudopapilloma of the pancreas. In this group, there were 6 cases of postoperative complications, and the complication rate was 40%. The main complications included: biochemical fistula in 2 cases (13.3%), grade B pancreatic fistula in 1 case (6.7%), gastric emptying disorder in 2 cases (13.3%), abdominal infection in 1 case (6.7%), diarrhea in 1 case (6.7%), abdominal bleeding in 1 case (6.7%), portal vein thrombosis in 1 case (6.7%).

#### Long-term follow-up outcomes

**Figure 4:**
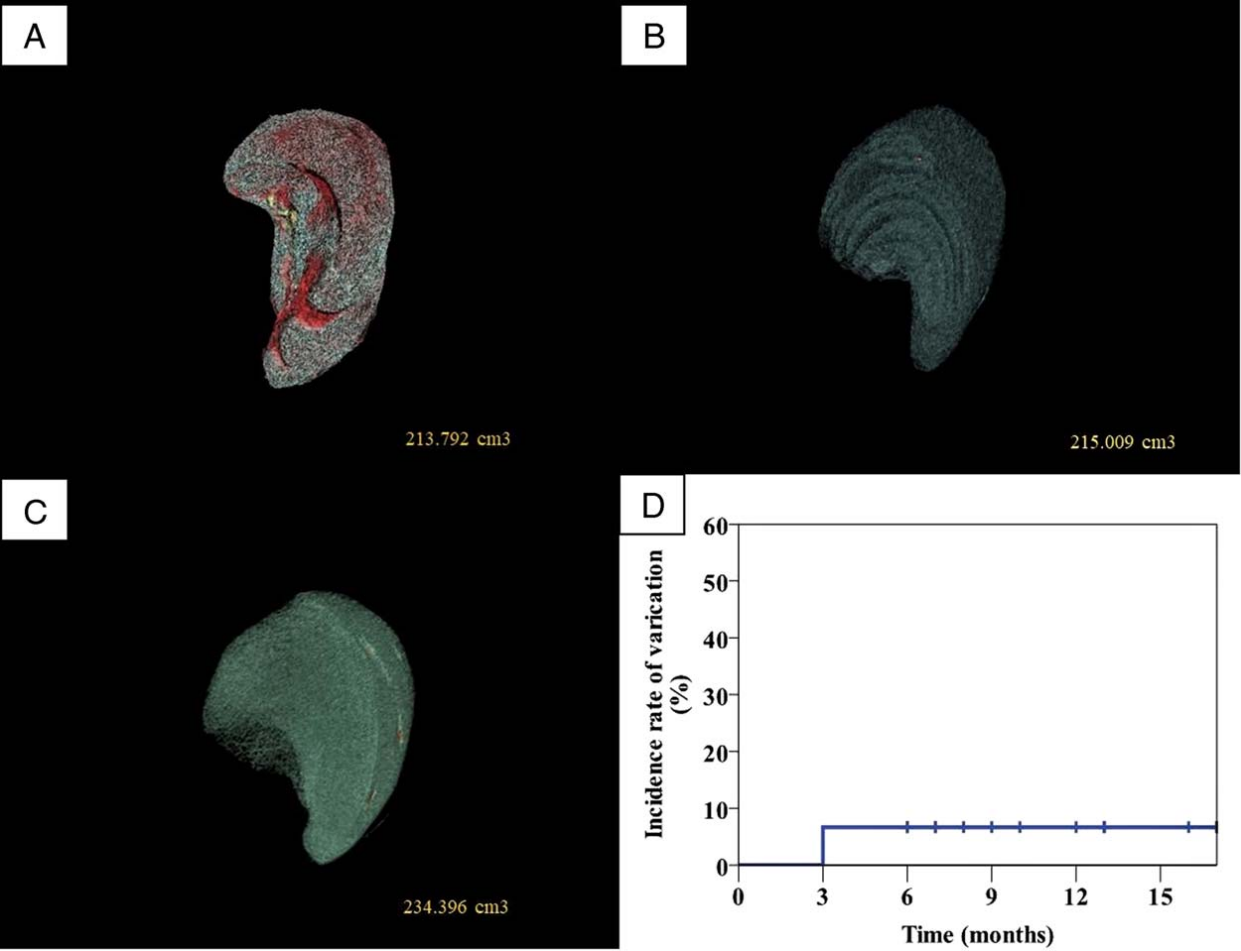
Postoperative follow-up of patients undergoing portal vein system reconstruction (*n*=15). (A) Three-dimensional reconstruction of the patient’s spleen volume before surgery (No.3 patient). (B) Three-dimensional reconstruction of spleen volume in 1 month after surgery (No.3 patient). (C) Three-dimensional reconstruction of spleen volume in 6 month after surgery (No.3 patient). (D) Curve of varicose vein occurrence in all patients.

The last follow-up was December 2023, with a median follow-up of 12 months. The primary endpoint was varicose veins or death. During follow-up, one patient was found with EGV in the third month after surgery, which was considered to be related to the perioperative development of portal vein thrombosis, which led to SV stenosis. The postoperative platelets, platelet ratio, spleen volume, spleen volume ratio and varicose veins in this group were detailed in Table 6 and Figure 4. The results showed that after the reconstruction of the SV, there was no significant difference in the proportion of platelet count and platelet count after surgery, while the proportion of spleen volume and spleen volume increased slowly, and the incidence of SPH within 6 months after surgery was 6.7%.

**Table 6:** Dynamic changes in long-term follow-up indicators of patients (*n*=15).

Item	Preoperative	1 month postoperative	3 months postoperative	6 months postoperative
Platelets (×10^9^/l)	245.5±98.9	227.4±77.6	213.7±66.6	211.5±69.4
Platelet ratio (%)	100	94.7±13.2	91.0±22.4	90.±22.9
Spleen volume (ml)	175 (155, 232)	204 (169, 265)	207 (172, 388)^a^	202 (176, 416)^a^
Spleen volume ratio (%)	100	105.7 (102.2, 109.9)^a^	110.3 (103.4, 111.5)^a^	109.2 (105.9, 112.8)^a^

^a^ Indicates *P*<0.05 compared to preoperative period.

## Discussion

Although new progress and breakthroughs have been made in the treatment of various solid neoplasms in recent years, the prognosis of patients with pancreatic cancer remains very poor, the overall rate of radical resection (R0) is less than 30% meanwhile the overall 5-year survival rate is less than 10%^25^. According to the latest international statistics, the incidence of pancreatic cancer in the world is the 14^th^ malignant tumor; however, the death rate is the 6^th^ ^26^. At the same time, the literature indicates that 17–32% of pancreatic cancer patients have already had portal system (PV, SMV and SV) invasion when diagnosed^27^. SMV and PV invasion is frequent due to the proximity of these vessels to the uncinate process and pancreatic head. With the aim of obtaining a margin-free resection, PD with an “en-bloc” resection of the S-M-P confluence is well accepted for patients diagnosed as borderline resection or local advanced pancreatic cancinoma with infiltration of confluence and is believed to have survival benefits without increasing postoperative morbidity and mortality^28–31^. SV sometimes should be ligated during the operation procedures. Unfortunately, obstruction of venous reflux induced by ligation of SV may result in SPH, which remains to be a clinical syndrome due to outflow obstruction of SV, developing varices with hemorrhage and splenomegaly with thrombocytopenia^32^. When pancreatic cancer invades the S-M-P confluence, resection and reconstruction of the invaded vessels and ligation of SV are performed, after that, the incidence of SPH is about 67.2%. At the same time, active ligation of splenic artery (SA) may reduce the occurrence of SPH (38.5%), to some extent, although the incidence of SPH is rare, it is known as a life-threatening cause of upper gastrointestinal bleeding. SPH has become a critical problem after PD with resection of S-M-P confluence till now^12^.

Several clinical issues and reconstruction techniques have been described after resection of S-M-P confluence^10,33,34^. Some surgeons reported that there was no need for reconstruction of the SV and there were no serious complications after SV ligation, for instance, Tanaka H and his colleagues^13^ suggested that PD with S-M-P confluence resection without SV reconstruction could be safely conducted, preservation of left gastric vein-PV and/or IMV-SV confluences might reduce the risk of SPH, whereas most surgeons had different views and they performed various reconstruction methods to avoid or lessen SPH^9,35^.

Ferreira and colleagues^36^ reported that the incidence of SPH could be significantly decreased with SV-IMV anastomosis after resection or preservation of the natural SV-IMV confluence. However, Ono *et al.* ^9^ pointed out that since the blood flow from the spleen was the same when IMV was isolated, SV-IMV anastomosis or preservation of the natural SV-IMV confluence could not prevent the occurrence of SPH, the diagnosis of SPH should be considered in patients with upper gastrointestinal bleeding with splenomegaly and normal liver function. However, the most common form of SPH is asymptomatic and often incidentally detected during examination^7^. The first clinical manifestation of SPH is usually acute (usually massive) or chronic upper gastrointestinal bleeding caused by esophageal or gastric varices, rarely from colonic varices^37^. This is consistent with our results. In our study, the number of patients with esophageal and gastric varices appears much higher than that of colonic varices.

The alternatives for vascular reconstruction mainly include autologous vessels, allogeneic vessels and artificial vessels. At present, the reports of autologous vessels mainly include jugular vein, right ovarian vein, femoral vein, great saphenous vein, left renal vein, SV and so on^38^. However, the removal of autologous vessels is bound to bring new damage to surgical patients. Advanced pancreatic surgery centers at home and abroad mainly use artificial vessels for replacement, and in terms of artificial blood vessels, the existence of foreign bodies will also increase the infection rate and the incidence of vascular thrombosis. More importantly, due to the limitation of the shape of artificial vessels, for patients with invasion of S-M-P confluence, only SMV and PV can be reconstructed, SV often requires forced ligation. As we have mentioned before, this approach will lead to postoperative SPH, resulting in esophageal and gastric varices bleeding and thrombocytopenia caused by hypersplenism.

Based on years of experience in pancreatic cancer surgery, our center creatively put forward the classification of pancreatic cancer with vascular invasion and the specific surgical management strategies for various types^3,39^. On this basis, our center routinely chooses allogeneic vascular replacements for pancreatic cancer with vascular invasion. This not only avoids the surgical injury of autologous vessels, but also the allogeneic vessels themselves belong to the human body, and the tissue and mechanical structure are exactly the same as those of the patient. What is more advantageous is that the allogeneic vessels can be properly trimmed and reshaped according to the scope and shape of vascular defects, so as to maximize the original hemodynamic state and ensure the smooth recovery of organ perfusion and visceral function. The allogeneic iliac vessels were used in our center, and the natural bifurcations of the internal and external iliac vessels were used to form a Y shape. The common iliac vein was anastomosed with the PV, the external iliac vein was anastomosed with the SMV, and the internal iliac vein was anastomosed with the SV. Or extensive PD with S-M-P confluence resection can be successfully completed by using the allogeneic bifurcation of PV, SMV and SV. Reconstruction of the S-M-P confluence by bifurcated allogeneic vein, which is rarely reported, has its own advantages. The biggest advantage is that it can help to restore the normal anatomical structure and the fluid dynamics to the greatest extent because of its natural bifurcations. We confirmed that venous reconstruction by allogeneic vein is feasible and safety through close observation. Compared to SV simply ligation, reconstruction by allogeneic vein had comparable intraoperative blood loss and blood transfusion cases although longer operation time, and similar incidences of total complications and pancreatic fistula. Vascular-related complications such as venous thrombus, infection and graft rejection are not detected. In this study, we found that the incidence of left-sided portal hypertension was significantly reduced in patients underwent reconstruction by bifurcated allogeneic vein after venous resection. Platelet count had not obvious change before and after the operation, and varices was not found. The spleen volume ratio slightly increased at 6 months after operation. Through analysis, we found that allogeneic veins with a smaller caliber relative to their own were provided for a few patients. Unmatched vascular caliber might be the main cause for this phenomenon of a slightly increased spleen volume ratio.

The study also had some limitations. First, this is a single-center study with a small sample size, which may limit the generalizability of the findings. Large multicenter prospective studies are needed in the future to validate these results and ensure broader applicability.

Secondly, the use of allogeneic vascular replacement, specifically bifurcated allogeneic vessel replacement, is a unique feature of our center. As this technique is both novel and exclusive to our practice, developing a feasible research plan to promote its application across multiple global centers is necessary.

Thirdly, for the subset of locally advanced pancreatic cancer patients with longer survival periods and those requiring PD due to confluence invasion, future prospective studies should extend the follow-up period. This would provide more comprehensive clinical data, laying a solid foundation for the promotion and development of this technique.

## Conclusions

Without increased postoperative complications, reconstruction of the S-M-P confluence by bifurcated allogeneic vein after resection can avoid SPH satisfactorily. By demonstrating the efficacy and safety of this novel technique, reconstruction by bifurcated allogeneic vein may be the best method to prevent SPH after resection of the S-M-P confluence in patients with pancreatic cancer in the near future.

## Ethical approval

This prospective study was approved and implemented by the ethics committee of Beijing Chaoyang Hospital in January 2020 (No. 2021-Ke-261), and registered through the website of the China Clinical Trials Registry (No. ChiCTR2100049076), and retrospective study was approved by the ethics committee and the clinical application management committee of medical technology of Beijing Chaoyang Hospital for clinical application (No. 2020-D-301).

## Consent

Written informed consent was obtained from the patient for publication of this case report and accompanying images. A copy of the written consent is available for review by the Editor-in-Chief of this journal on request.

## Source of funding

Beijing Natural Science Foundation, Grant No. 7222303.

## Author contribution

Conception and design: J.W., S.-C.L., S.C. Administrative support: B.H., Q.H., R.L. Provision of study materials or patients: S.-C.L., R.L. Collection and assembly of data: J.-C.H., H.-X.W., J.W. Data analysis and interpretation: S.-C.L., J.W., S.-P.C. Manuscript writing: all authors. Final approval of manuscript: all authors. All authors made substantial contributions to conception and design, acquisition of data, or analysis and interpretation of data; took part in drafting the article or revising it critically for important intellectual content; agreed to submit to the current journal; gave final approval of the version to be published; and agree to be accountable for all aspects of the work.

## Conflicts of interest disclosure

The authors declare that there has no conflict of interest.

## Research registration unique identifying number (UIN)

website of the China Clinical Trials Registry (No. ChiCTR2100049076).

## Guarantor

Ren Lang.

## Data availability statement

The data used and analyzed in this study are included in the article or are available from the corresponding and first authors on reasonable request.

## Provenance and peer review

Not commissioned, externally peer-reviewed.

## Presentation (for original articles only)

None.

## Supplementary Material
