# Supplementary material for: Utilizing bifurcated allogeneic vein grafts: a novel approach for preventing sinistral portal hypertension following pancreaticoduodenectomy. A 10-year before and after study
Source: Int J Surg. 2024 Jul 12;111(1):9–19. doi: 10.1097/JS9.0000000000001944 (PMC11745578; doi:10.1097/JS9.0000000000001944)
Supplement: Supplementary file 1 [file js9-111-0009-s001.pdf]

The STROCSS Guideline		
**Item no.**	**Item description**	**Page No.**
**TITLE**		
1	Title:<br>- The word cohort or cross-sectional or case-controlled is included<br>- The area of focus is described (e.g. disease, exposure/intervention, outcome)<br>- Key elements of study design are stated (e.g. retrospective or prospective)	1
**ABSTRACT**		
2a	Introduction: the following points are briefly described<br>- Background<br>- Scientific Rationale for this study	1
2b	Methods: the following areas are briefly described<br>- Study design (cohort, retro-/prospective, single/multi-centred)<br>- Patient populations and/or groups, including control group, if applicable<br>- Interventions (type, operators, recipients, timeframes)<br>- Outcome measures	1
2c	Results: the following areas are briefly described<br>- Summary data (with statistical relevance) with qualitative descriptions, where appropriate	1
2d	Conclusion: the following areas are briefly described<br>- Key conclusions<br>- Implications to practice<br>- Direction of and need for future research	1
**INTRODUCTION**		
3	Introduction: the following areas are described in full<br>- Relevant background and scientific rationale<br>- Aims and objectives<br>- Research question and hypotheses, where appropriate	2-3
**METHODS**		
4a	Registration and ethics<br>- Research Registry number is stated, in accordance with the declaration of Helsinki*<br>- All studies (including retrospective) should be registered before submission<br><br>*"*Every research study involving human subjects must be registered in a publicly accessible database before recruitment of the first subject*" (this can be obtained from; ResearchRegistry.com or ClinicalTrials.gov or ISRCTN)	4
4b	Ethical Approval: the following areas are described in full<br>- Necessity for ethical approval<br>- Ethical approval, with relevant judgement reference from ethics committees<br>- Where ethics was unnecessary, reasons are provided	4
4c	Protocol: the following areas are described comprehensively<br>- Protocol (*a priori* or otherwise) details, with access directions<br>- If published, journal mentioned with the reference provided	5

4d	Patient involvement in Research<br>- Describe how, if at all, patients were involved in study design e.g. were they involved on the study steering committee, did they provide input on outcome selection, etc.	5-6
5a	Study Design: the following areas are described comprehensively<br>- 'Cohort' study is mentioned<br>- Design (e.g. retro-/prospective, single/multi-centred)	4-6
5b	Setting: the following areas are described comprehensively<br>- Geographical location<br>- Nature of institution (e.g. academic/community, public/private)<br>- Dates (recruitment, exposure, follow-up, data collection)	4-6
5c	Cohort Groups: the following areas are described in full<br>- Number of groups<br>- Division of intervention between groups	4-6
5d	Subgroup Analysis: the following areas are described comprehensively<br>- Planned subgroup analyses<br>- Methods used to examine subgroups and their interactions	-
6a	Participants: the following areas are described comprehensively<br>- Eligibility criteria<br>- Recruitment sources<br>- Length and methods of follow-up	5-6
6b	Recruitment: the following areas are described comprehensively<br>- Methods of recruitment to each patient group<br>- Period of recruitment	4-6
6c	Sample Size: the following areas are described comprehensively<br>- Margin of error calculation<br>- Analysis to determine study population<br>- Power calculations, where appropriate	4-6
**Intervention and Considerations**		
7a	Pre-intervention Considerations: the following areas are described comprehensively<br>- Patient optimisation (pre-surgical measures)<br>- Pre-intervention treatment (hypothermia/-volaemia/-tension; ICU care; bleeding problems; medications)	4-6
7b	Intervention: the following areas are described comprehensively<br>- Type of intervention and reasoning (e.g. pharmacological, surgical, physiotherapy, psychological)<br>- Aim of intervention (preventative/therapeutic)<br>- Concurrent treatments (antibiotics, analgaesia, anti-emetics, NBM, VTE prophylaxis)<br>- Manufacturer and model details where applicable	4-6
7c	Intra-Intervention Considerations: the following areas are described comprehensively<br>- Administration of intervention (location, surgical details, anaesthetic, positioning, equipment needed, preparation, devices, sutures, operative time)<br>- Pharmacological therapies include formulation, dosages, routes and durations<br>- Figures other media are used to illustrate	4-6

7d	Operator Details: the following areas are described comprehensively<br>- Training needed<br>- Learning curve for technique<br>- Specialisation and relevant training	4-6
7e	Quality Control: the following areas are described comprehensively<br>- Measures taken to reduce variation<br>- Measures taken to ensure quality and consistency in intervention delivery	4-6
7f	Post-Intervention Considerations: the following areas are described comprehensively<br>- Post-operative instructions and care<br>- Follow-up measures<br>- Future surveillance requirements (e.g. imaging, blood tests)	4-6
8	Outcomes: the following areas are described comprehensively<br>- Primary outcomes, including validation, where applicable<br>- Definitions of outcomes<br>- Secondary outcomes, where appropriate<br>- Follow-up period for outcome assessment, divided by group	4-6
9	Statistics: the following areas are described comprehensively<br>- Statistical tests, packages/software used, and interpretation of significance<br>- Confounders and their control, if known<br>- Analysis approach (e.g. intention to treat/per protocol)<br>- Sub-group analysis, if any	7
**RESULTS**		
10a	Participants: the following areas are described comprehensively<br>- Flow of participants (recruitment, non-participation, cross-over and withdrawal, with reasons)<br>- Population demographics (prognostic features, relevant socioeconomic features, and significant numerical differences)	-
10b	Participant Comparison: the following areas are described comprehensively<br>- Table comparing demographic included<br>- Differences, with statistical relevance<br>- Any group matching, with methods	8-10
10c	Intervention: the following areas are described comprehensively<br>- Changes to interventions, with rationale and diagram, if appropriate<br>- Learning required for interventions<br>- Degree of novelty for intervention	8-10
11a	Outcomes: the following areas are described comprehensively<br>- Clinician-assessed and patient-reported outcomes for each group<br>- Relevant photographs and imaging are desirable<br>- Confounders to outcomes and which are adjusted	8-10
11b	Tolerance: the following areas are described comprehensively<br>- Assessment of tolerance<br>- Loss to follow up, with reasons (percentage and fraction)<br>- Cross-over with explanation	8-10
11c	Complications: the following areas are described comprehensively<br>- Adverse events described<br>- Classified according to Clavien-Dindo classification*	8-10

	- Mitigation for adverse events (blood loss, wound care, revision surgery should be specified)<br><br>*Dindo D, Demartines N, Clavien P-A. Classification of Surgical Complications. A New Proposal with Evaluation in a Cohort of 6336 Patients and Results of a Survey. Ann Surg. 2004; 240(2): 205-213	
12	Key Results: the following areas are described comprehensively<br>- Key results, including relevant raw data<br>- Statistical analyses with significance	8-10
**DISCUSSION**		
13	Discussion: the following areas are described comprehensively<br>- Conclusions and rationale<br>- Reference to relevant literature<br>- Implications to clinical practice<br>- Comparison to current gold standard of care<br>- Relevant hypothesis generation	11-13
14	Strengths and Limitations: the following areas are described comprehensively<br>- Strengths of the study<br>- Limitations and potential impact on results<br>- Assessment of bias and management	11-13
15	Implications and Relevance: the following areas are described comprehensively<br>- Relevance of findings and potential implications to clinical practice are detailed<br>- Future research that is needed is described, with study designs detailed	11-13
**CONCLUSION**		
16	Conclusions:<br>- Key conclusions are summarised<br>- Key directions for future research are summarised	14
**DECLARATIONS**		
17a	Conflicts of interest<br>- Conflicts of interest, if any, are described	15
17b	Funding<br>- Sources of funding (e.g. grant details), if any, are clearly stated	15
