# Supplementary material for: Utilizing bifurcated allogeneic vein grafts: a novel approach for preventing sinistral portal hypertension following pancreaticoduodenectomy. A 10-year before and after study
Source: Int J Surg. 2024 Jul 12;111(1):9–19. doi: 10.1097/JS9.0000000000001944 (PMC11745578; doi:10.1097/JS9.0000000000001944)
Supplement: Supplementary file 2 [file js9-111-0009-s002.docx]

**Intraoperative Vascular Replacement Procedure:**

First, use vascular clamps to respectively block the PV (portal vein), SMV (superior mesenteric vein), and SV (splenic vein). Then, sever the vessels more than 5mm from the tumor invasion margin, and excise the tumor along with the invaded vessels en bloc. Send the intraoperative frozen sections of the three severed vessels for pathological margin assessment.. Meanwhile, take the cryopreserved allogeneic vessels and tailor them according to the vascular defect ex vivo (S-Figure 1A-C). After confirming negative margins from the intraoperative pathology analysis, proceed with the reconstruction using the allogeneic vessels.

To alleviate intestinal congestion and quickly restore intestinal blood flow, we prioritize the reconstruction of the SMV. Using 6-0 prolene sutures, perform continuous everting anastomosis of the anterior and posterior walls between the allogeneic vessel and the SMV. Then, perform similar anastomosis between the allogeneic vessel and the PV. Release the vascular clamps on the PV and SMV to restore blood flow between them. Subsequently, perform continuous everting anastomosis of the anterior and posterior walls between the allogeneic vessel and the SV using 6-0 prolene sutures. Release the clamp on the SV to restore blood flow between the SV and the PV, thus completing the allogeneic vessel replacement (S-Figure 1D).

**Acquisition of Allogeneic Vessels:**

1. Source of Allogeneic Vessels:

Allogeneic vessels are primarily sourced from adult brain-dead organ donors who meet the following criteria:

1. Female donors under 60 years old and male donors under 55 years old; (2) No history of atherosclerosis or other cardiovascular diseases; (3) No history of tumors or infectious diseases; (4)The harvested vessels should not exhibit atherosclerosis and/or calcification, luminal stenosis, ulcerative lesions, aneurysmal dilation, severe hematomas or infections of the vessel wall, or incomplete vessel walls; (5)Warm ischemia time should not exceed 6 hours, cold ischemia time should not exceed 24 hours, and the time from vessel harvest to cryopreservation should not exceed 72 hours; (6) Vascular tissue donation and harvesting must comply with ethical principles.
2. Pre-preservation Processing of Allogeneic Vessels:

After harvesting the allogeneic vessels, perform appropriate trimming to remove surrounding fat, nerve fibers, and other tissues. Flush the lumen and periphery with heparinized saline to prevent thrombus formation. Conduct morphological evaluation, measuring and recording the length, branches, and diameter of the vessels. Incubate the vessels in an antibiotic-medium mixture containing vancomycin and amikacin at 2-8°C for 24 hours. After incubation, rinse the vessels with saline and proceed with the appropriate preservation method. Label the vessels with the donor’s blood type and the preservation date. Send a portion of the vascular tissue for microbiological culture to ensure safety before preservation.

1. Preservation Method of Allogeneic Vessels:

We use low-temperature preservation, which involves storing the allogeneic vessels in UW solution at 4°C. Vessels preserved using this method are relatively safe and effective for up to 2 weeks. Before use, retrieve the allogeneic vessels and allow them to reach room temperature (15-20 minutes), then repeatedly rinse with saline and place them in heparinized saline for use.


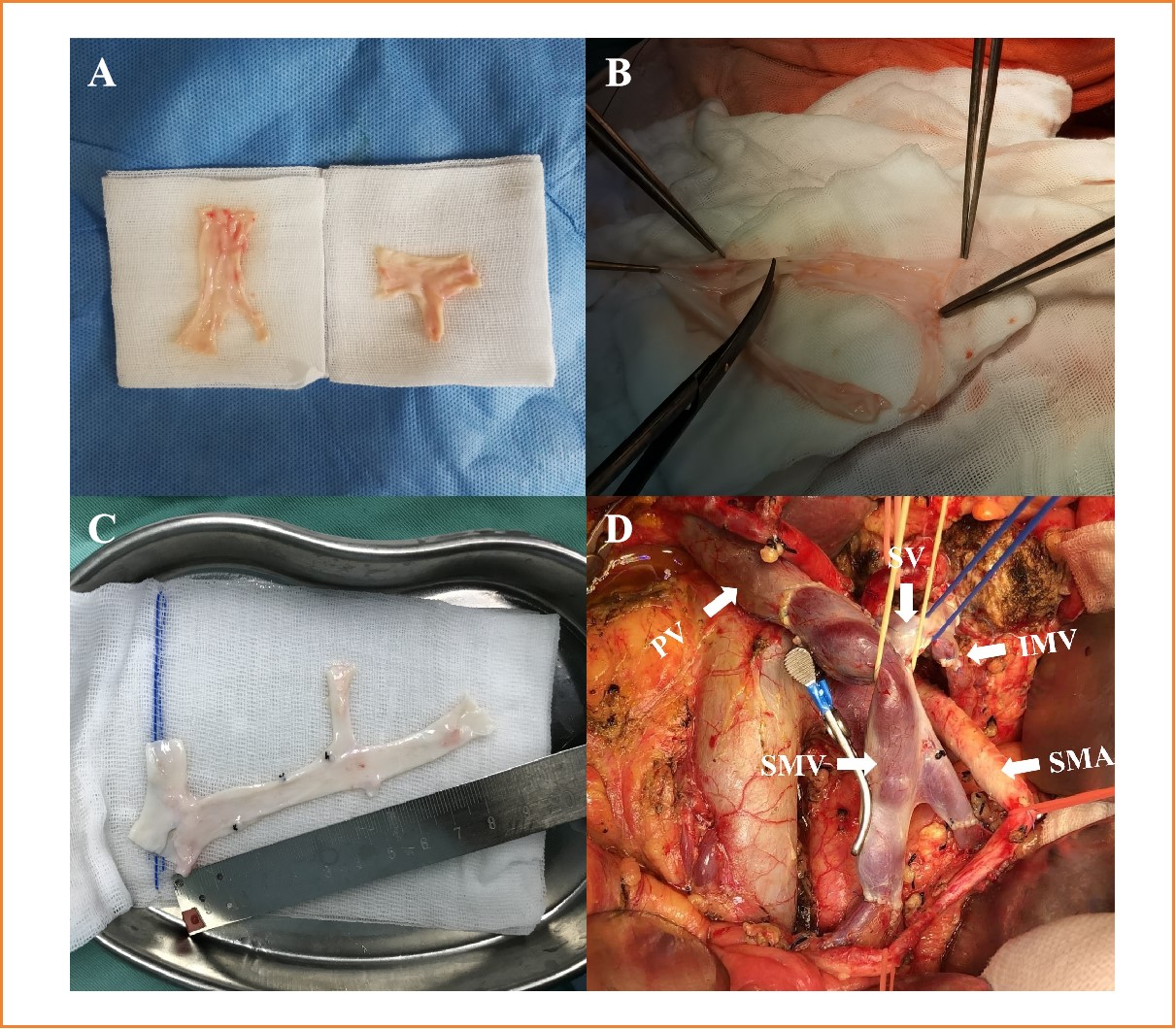


1. Figure 1.
2. C:Preparation Method and Ex Vivo Morphology of Bifurcated Allogeneic Vessels.

D: Illustration of Complete Portal Venous System Replacement.
